# L2 Vocabulary Learning Through Virtual Reality: Effects of Affective and Cognitive Variables

**DOI:** 10.3390/bs16101939

**Published:** 2026-10-19

**Authors:** Hui Jiang, Guanyu Zhou, John W. Schwieter, Lu Jiao

**Affiliations:** 1 Office of Discipline Development, Qingdao University, Qingdao 266071, China; 2 School of Education Science, Qingdao University, Qingdao 266071, China; 3 Language Acquisition, Multilingualism, and Cognition Laboratory/Bilingualism Matters, Wilfrid Laurier University, Waterloo, ON N2L 3C5, Canada; 4 Department of Linguistics and Languages, McMaster University, Hamilton, ON L8S 4L8, Canada; 5 School of Education, University College Dublin, D04 C1P1 Dublin, Ireland; 6 Brain Cognition and Language Learning Laboratory, Qingdao University, Qingdao 266071, China

**Keywords:** virtual reality, L2 vocabulary learning, cognitive-affective effects

## Abstract

Immersive virtual reality (IVR) has shown positive effects on second language (L2) learning, yet the mechanisms underlying these effects remain unclear. Based on the Cognitive Affective Model of Immersive Learning (CAMIL), the present study used structural equation modeling to examine how affective (situational interest, intrinsic motivation, emotions) and cognitive variables (cognitive load, embodied learning, self-efficacy) contribute to receptive L2 vocabulary learning through IVR. A group of native Chinese speakers was asked to learn Korean words in an IVR environment. They were then administered a lexical recognition task that measured learning outcomes. The results revealed two sequences of associations through which IVR affected vocabulary recognition performance: a cognitive path that intersected with presence and self-efficacy; and an affective path that intersected with presence and emotions. Situational interest and intrinsic motivation also demonstrated a sequence of associations with receptive L2 vocabulary recognition. Although further independent confirmation is needed, these findings put forth theoretically meaningful patterns regarding the cognitive-affective mechanisms of IVR-based L2 learning.

## 1. Introduction

Vocabulary learning is foundational in mastering a second language (L2) (Stoffelsma et al., 2020). However, it poses a significant challenge for many individuals, particularly for adults (Lan, 2013; Meara, 2008). Various digital technologies have been employed to promote L2 vocabulary learning. For instance, prior research has explored real-world pervasive digital environments, demonstrating the crucial role of multisensory engagement in language learning (Pallotti et al., 2017; Park & Seedhouse, 2017). More recently, immersive virtual reality (IVR) has further advanced this paradigm by offering new avenues for embodied L2 vocabulary learning (Jiao et al., 2026; Liu et al., 2026). Despite accumulating evidence that IVR environments can benefit L2 vocabulary learning, prior work has primarily examined the outcomes of L2 vocabulary learning (Fuhrman et al., 2020; Jiao et al., 2024; Legault et al., 2019). It remains unclear as to how IVR contributes to the process of L2 vocabulary learning, an issue which we explore in the present study.

### 1.1. L2 Vocabulary Learning Through IVR

Immersive virtual reality (IVR) is increasingly recognized as a promising tool for both L2 vocabulary learning (Jiao et al., 2024; Legault et al., 2019; Liu et al., 2024; Liu et al., 2026) and science learning (Klingenberg et al., 2024; Parong & Mayer, 2018), as it offers on-demand, contextual support (Li & Jeong, 2020). Growing evidence highlights the beneficial effects of IVR environment on L2 vocabulary learning outcomes (Legault et al., 2019; Jiao et al., 2024, 2026). For example, in the study by Legault et al. (2019), English monolinguals learned Chinese words in either an IVR context or through text translations (i.e., word-word pairs). Results from the study indicated that participants learned new words better in the IVR condition compared to word-word pairs, showing the effectiveness of using IVR in L2 learning. Similarly, more recent studies have reported positive effects of IVR on both the learning of lexical forms and the semantic integration of novel words (Jiao et al., 2024, 2026).

However, as mentioned above, this body of work has largely examined the outcomes of vocabulary learning rather than the cognitive and affective processes underlying how such learning unfolds. To the best of our knowledge, only a few studies have focused on how IVR affects the processes of L2 vocabulary learning (C. Chen & Yuan, 2025; Y. J. Lin & Wang, 2021). For instance, Y. J. Lin and Wang (2021) found that IVR facilitated learners’ self-efficacy and motivation during the L2 vocabulary learning process. Nonetheless, it remains unknown how the cognitive and affective variables interact with each other while learning, as well as how they contribute to learning outcomes. Building on the Cognitive Affective Model of Immersive Learning (CAMIL; Makransky & Petersen, 2021), the present study used structural equation modeling (SEM) to test cognitive and affective variables that may influence L2 vocabulary learning through IVR simulation.

### 1.2. Theoretical Framework

**Figure 1:**
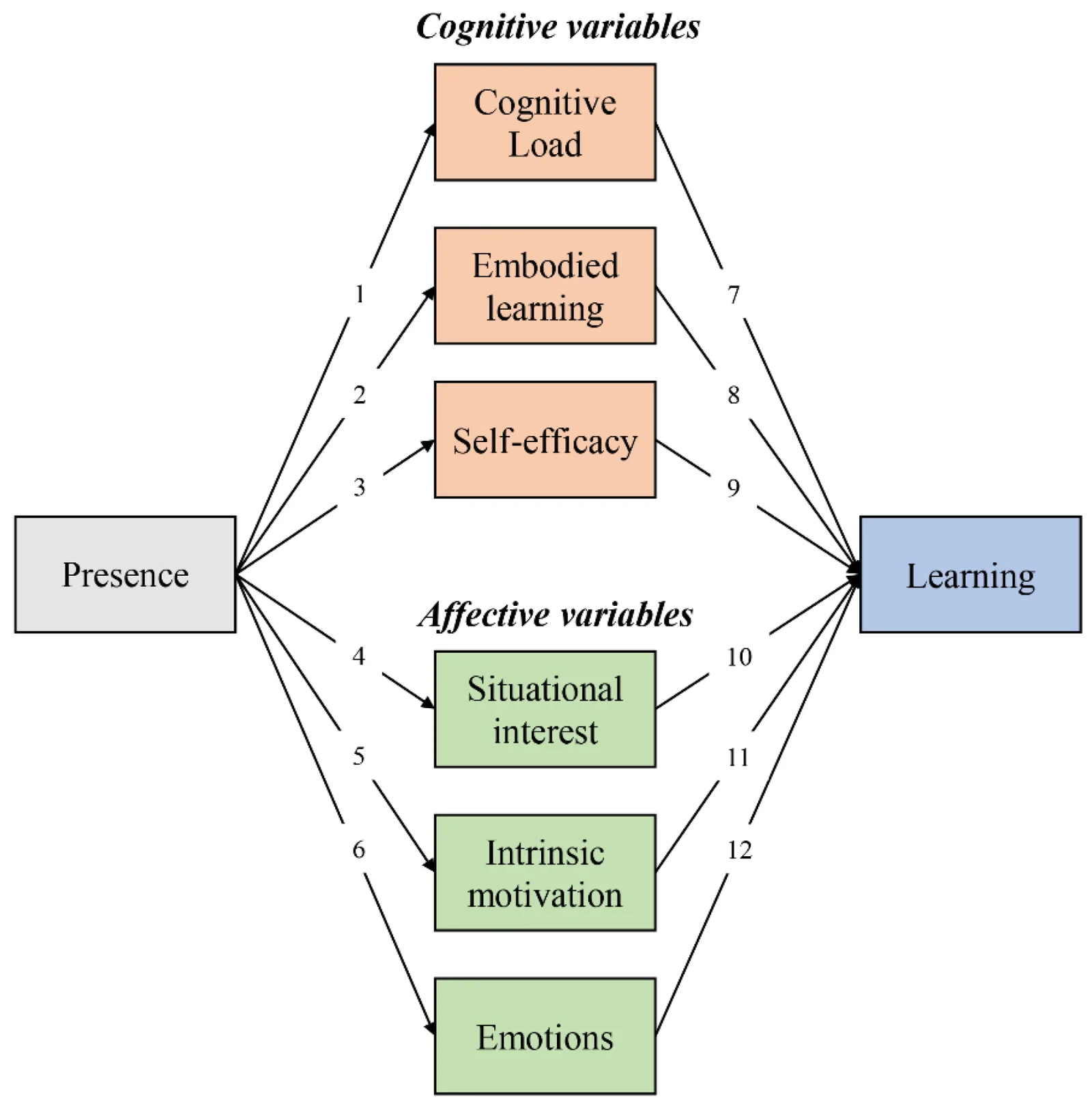
The present study’s hypothesized model based on the Cognitive Affective Model of Immersive Learning (CAMIL). The numbers denote the specific directional paths corresponding to the research hypotheses described in Section 1.4.

The a priori research model hypothesized and tested in the present study (see Figure 1) builds on the Cognitive Affective Model of Immersive Learning (CAMIL) framework. The framework proposes two possibilities regarding learning in immersive environments (Makransky & Petersen, 2021). First, the main affordance of learning is the higher sense of presence, that is, the psychological sense of ‘being there’ (see Section 1.3.1). The immersion principle in multimedia learning also proposes that more presence can lead to better learning outcomes when it is constructively used in designing a lesson (Makransky, 2021). Second, the immersive characteristics of IVR environment can influence several cognitive and affective variables that either facilitate or hinder learning outcomes (Makransky & Petersen, 2021). Therefore, the present study focuses on how the sense of presence in IVR contributes to L2 vocabulary learning by simultaneously examining the cognitive and affective paths underlying this process.

Although the CAMIL is based on empirical research, few empirical studies have thus far tested the paths outlined in the framework. To our knowledge, only one study has employed SEM analyses to test the CAMIL, and did so among participants who were engaged in a virtual science lesson on viral diseases (Petersen et al., 2022). It remains unclear whether the framework can be applied to L2 learning. In the current experiment, we draw on the CAMIL as a point of departure and apply SEM to examine the affective and cognitive factors (depicted in Figure 1) that play a role in immersive L2 vocabulary learning.

### 1.3. Variables Examined

#### 1.3.1. Presence

Presence refers to the sense of ‘being there’ and has been identified as a subjective measure of immersion (i.e., more immersive environments yield higher presence; Ijsselsteijn & Riva, 2003). Lee (2004) defines presence as a psychological state in which virtual objects are experienced as actual physical objects in sensory and/or non-sensory ways. According to the CAMIL (Makransky & Petersen, 2021), the main affordance of learning in immersive environments is a higher sense of presence. An immersive environment delivers rich and fully embodied experiences and creates strong feelings of presence, which in turn motivates learners to process information more deeply (e.g., Makransky & Lilleholt, 2018; Makransky et al., 2019).

#### 1.3.2. Cognitive Variables

The cognitive factors examined in the present study are cognitive load, embodied learning, and self-efficacy. Cognitive load refers to the working memory resources required for information processing of a particular task (Leppink et al., 2014; Paas et al., 2010; Sweller, 2011). IVR may provide extensive stimuli and lead to higher levels of cognitive load compared to other presentation formats (Parong & Mayer, 2021; Richards & Taylor, 2015). The second cognitive factor of interest is embodied learning, which refers to the notion that individuals come to know themselves and the world around them “as a ‘lived body’ subject that senses and does the sensing in a meaningful way” (Stolz, 2015, p. 483). Virtual reality fosters embodied learning by rendering modifiable virtual environments in which users’ actions are mapped onto a virtual body. The third cognitive factor is self-efficacy, i.e., people’s beliefs in their capability to attain goals. Empirical studies indicate that virtual reality can have beneficial effects on learners’ self-efficacy compared to learning through simple videos (Meyer et al., 2019) or text (Makransky et al., 2019).

#### 1.3.3. Affective Variables

The affective factors of interest in the present study include situational interest, intrinsic motivation, and emotions. Situational interest refers to the transient and context-specific engagement of heightened cognitive focus, attention, and curiosity in response to environmental stimuli (Hidi & Renninger, 2006). According to the CAMIL, virtual reality provides an effective way of triggering and maintaining situational interest (see Makransky et al., 2020b). The second affective variable is intrinsic motivation, which occurs when an individual engages in an activity because of the inherent satisfaction it gives them as opposed to doing so for external reasons (Ryan & Deci, 2000). Randomized, controlled research comparing virtual conditions to other presentation formats indicates that the former leads to higher levels of intrinsic motivation than the latter (Makransky & Petersen, 2019). Finally, the third affective factor, emotions, are feelings from judgments relative to specific events and can be intense and directed (Linnenbrink-Garcia et al., 2016). In the present study, we focus on the positive emotional experience of learning. Previous research has shown that virtual reality lessons may induce positive emotions which can facilitate learning, at least in the science domain (e.g., Makransky & Lilleholt, 2018; Parong & Mayer, 2021).

#### 1.3.4. Learning Outcomes

The learning outcomes (i.e., performance on L2 vocabulary learning in IVR) explored in this study were measured as accuracy and reaction times (RTs) through a lexical recognition task. We directly assess individuals’ ability to map novel L2 forms to visual concepts. RT was selected as a critical dependent variable, as it is a widely recognized index of lexical access, automaticity, and learning efficiency in L2 learning research (Y. Lin et al., 2026).

### 1.4. Hypotheses

The predicted relationships in the a priori model are presented in Figure 1. Based on the CAMIL, we propose the following hypotheses:

**Hypothesis** **1** **(paths** **1–3).** *Presence will predict cognitive variables. Specifically, higher presence will lead to increased embodied learning, boosting learners’ self-efficacy and influencing their cognitive load.*

**Hypothesis** **2** **(paths** **4–6).** *Presence will predict affective variables. Specifically, higher presence will trigger higher situational interest, foster intrinsic motivation, and elicit positive emotions.*

**Hypothesis** **3** **(paths** **7–9).** *Cognitive variables will predict outcome variables.*

**Hypothesis** **4** **(paths** **10–12).** *Affective variables will predict outcome variables.*

## 2. Methods

### 2.1. Participants

The sample consisted of 112 undergraduate students (78 women, 34 men; M_age_ = 20.33 ± 1.71). All participants were right-handed native Chinese speakers and reported no prior knowledge of Korean, the language in which the new words were learned in the experiment. Twelve participants were excluded from the analyses because their post-test accuracy was below 75%, leaving a final sample of 100 participants for the statistical analyses. The study was approved by the local research ethics board, and all participants provided their informed consent before participating.

### 2.2. Materials

The materials comprised 20 individual Korean words, 20 3D objects, and a virtual scene. The Korean words (e.g., 무) consisted of 4–7 strokes (M = 5.80, SD = 1.01). The pronunciation of the to-be-learned words was recorded by a highly proficient female Korean speaker. The rationale for choosing Korean as the target language is that all participants reported no prior knowledge or experience with Korean. Previous work examining Chinese-English bilinguals has also selected Korean as the target language in which 3D objects were randomly assigned to to-be-learned new words (Liu et al., 2024).

**Figure 2:**
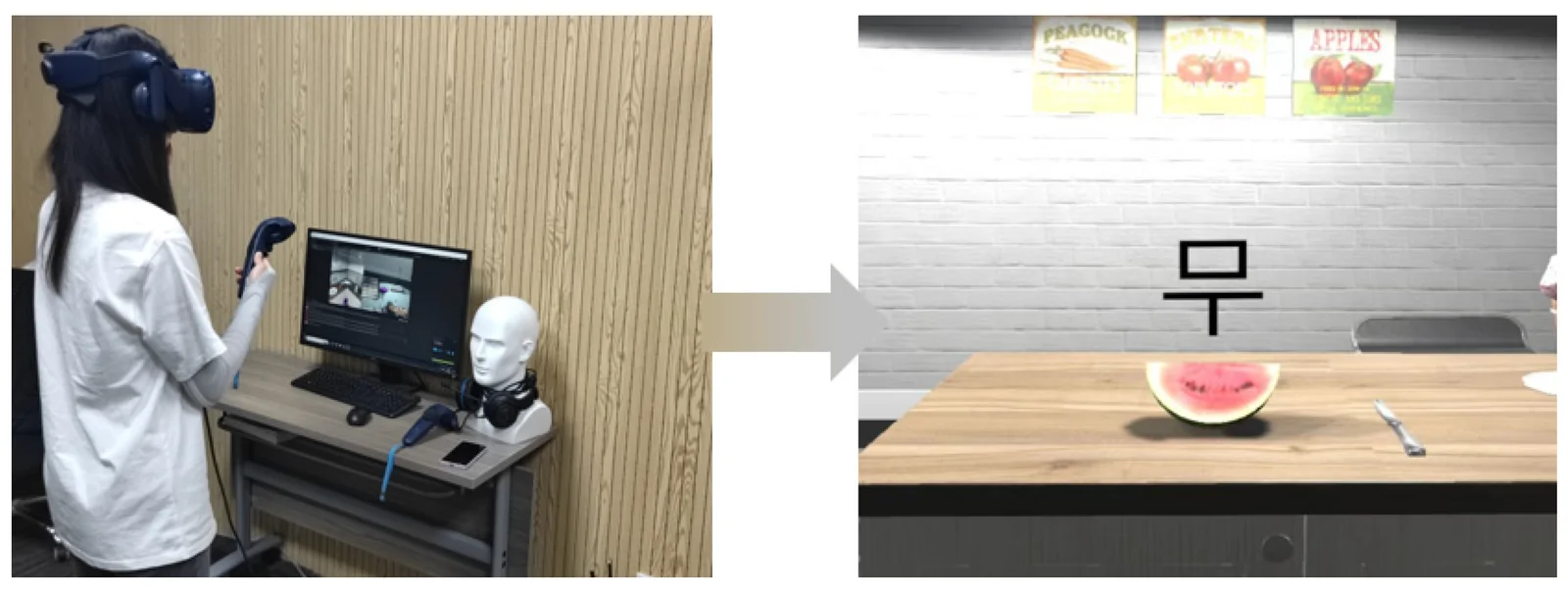
Screenshot of the virtual kitchen environment.

A virtual kitchen environment was created using the Unity software (Unity 6.0 LTS) and was used to simulate the learning context (see Figure 2). The 3D objects were chosen from a standardized database (Peeters, 2018) based on their familiarity, visual complexity, and suitability for integration into the virtual kitchen environment. A head-mounted display was used to present the virtual environment, fully immersing learners’ field of view and enabling stereoscopic and spatial perception of the virtual surroundings. By using a right-handed controller, participants had the ability to move freely between various positions of their choosing within the environment.

### 2.3. Procedure

The experiment consisted of three sessions and was conducted over two days. On Day 1, the participants completed a language background questionnaire and learned Korean words in an IVR environment, utilizing an HTC Vive (HTC Corporation, Taoyuan Taiwan) headset and handheld controllers. On Day 2, participants again learned the same Korean words using IVR, followed by a post-test. The learning duration was intentionally designed to provide sufficient time for participants to interact with the 3D objects so as to develop the intended cognitive and affective states.

#### 2.3.1. Pre-Test Session

In the pre-test session, participants were asked to complete a language background questionnaire in which they rated their language proficiency levels based on a 7-point scale (1 = very poor, 7 = excellent). Their ratings suggested that they were all native Chinese speakers.

#### 2.3.2. Learning Sessions

An IVR context provides learners with first-person, dynamic experiences that include opportunities to actively explore the surrounding environment, fostering an immersive experience (J. C. Chen, 2016). In the learning sessions, participants learned 20 Korean words in a simulated kitchen environment supported by VR technology. Before the learning session, the participants were shown how to use the VR equipment. After becoming familiar with the equipment, the participants maneuvered around and interacted with target objects in the virtual kitchen environment, using the handset to laser-point to and select 3D objects. Upon selecting the objects, they saw a target Korean word and heard its pronunciation. The first learning session on Day 1 lasted for 40 min, which consisted of 20 min of fixed-order learning and 20 min of free learning. During the fixed-order phase, the IVR system systematically presented these objects paired with their words one by one to ensure that all participants received an equal amount of baseline exposure to each word. Subsequently, in the free learning phase, participants were granted full autonomy to navigate throughout the virtual environment and use their hand-held controllers to select and review objects at their own pace and based on their individual needs. The second learning session on Day 2 lasted for 20 min, during which participants reviewed the words they learned on the first day.

#### 2.3.3. Post-Test Session

After the learning session on Day 2, participants were asked to perform a four-alternative forced-choice task, a lexical recognition test that estimated learning outcomes. In the task, each target word was presented with four pictures and participants were asked to select the correct picture that matched the word. Each trial began with a red fixation cross for 600 ms. Then, a target word was presented on the screen accompanied by four pictures, one of which was the target picture and three of which were non-target, interference pictures. Participants were required to select the correct picture of the target word within four seconds. Once a response was given by pressing a corresponding keyboard button, a blank screen was presented for 1000 ms prior to the next trial. Responses were scored as 1 or 0 depending on whether they did or did not match the correct answer, respectively. Before the formal experiment, the participants were presented with five practice trials to familiarize themselves with the procedure.

Following the recognition task, the participants answered a series of questions measuring physical presence, cognitive load, embodied learning, situational interest, intrinsic motivation, self-efficacy, and emotions. These questions were either the same as or revised from validated instruments in the previous literature (see Table A1 in Appendix A for full versions in English and Chinese). The original questions were in English and were translated into Chinese by a proficient Chinese-English bilingual. The translated questions were then back-translated by another proficient Chinese-English bilingual to ensure accuracy of the translation. The Chinese versions of both scales were controlled by one independent judge to ensure consistency with the original version.

The presence scale consisted of seven items adapted from Witmer and Singer (1998) and Makransky et al. (2017) (e.g., The virtual environment seemed real to me). The cognitive load scale included seven items from Leppink et al. (2014) (e.g., The learning content of this activity was very complex). The embodied learning scale comprised three items from Skulmowski and Rey (2018) (e.g., During the lesson, my movements and bodily activities were an integrated part of learning). The situational interest scale consisted of six items from Knogler et al. (2015) (e.g., Did the lesson capture your attention?). The intrinsic motivation scale included five items adapted from Makransky and Petersen (2019) and Parong and Mayer (2021) (e.g., It’s fun to learn vocabulary in a virtual reality scenario). The self-efficacy scale contained four items adapted from Meyer et al. (2019) and Pintrich et al. (1991) (e.g., I’m confident I could do an excellent job on vocabulary learning). Finally, emotions consisted of five items based on Parong and Mayer (2018) (e.g., I felt happy during the lesson).

#### 2.3.4. Statistical Analyses

We used SEM to test our hypotheses. SEM involves a two-step approach. The first step tests the measurement model through a confirmatory factor analysis, ensuring that specific items validly measure the latent psychological constructs. The second step tests the structural model by examining the hypothesized directional paths.

All statistical analyses were conducted with Mplus 8.3 software. The maximum likelihood method was chosen as the estimation method for further analysis. A *p*-value < 0.05 was considered statistically significant. In the SEM, five common goodness-of-fit indices were used to assess how well the model represented the data: the relative χ^2^/*df*, root mean square error of approximation (RMSEA), Comparative Fit Index (CFI), Tucker–Lewis Index (TLI), and standardized root mean square residual (SRMR). The desired values were: χ^2^/*df* < 3 (Bagozzi & Yi, 1988), RMSEA < 0.08 (Sharma et al., 2005), CFI and TLI ≥ 0.90, and SRMR < 0.08 (Hu & Bentler, 1999).

Accuracy rates and RTs were both analyzed; however, given that none of the cognitive and affective variables predicted accuracy of learning outcomes (*p*s > 0.05), we only report the results of the RT analyses (i.e., recognition speed of newly learned Korean words). In these analyses, incorrect trials and trials that were ±2.5 SDs from the mean were excluded (2.9%). RTs have consistently been used as a behavioral indicator of learning efficiency in the literature on L2 learning (see Jiao et al., 2026; Liu et al., 2024).

## 3. Results

### 3.1. Quality of Measures

For all measures except learning, Cronbach’s alpha was used as an indicator of scale quality. As recommended by DeVellis (2012), we considered all alpha levels between 0.80 and 0.90 as indicative of scale reliability. All constructs had Cronbach’s α values ≥ 0.80 (see Table 1).

A confirmatory factor analysis was conducted to assess the quality of the measurement model for the remaining 100 data points. In accordance with Hair et al. (2009), six items were removed to improve psychometric properties. Specifically, two items (‘E_4–E_5’) were excluded due to unacceptably low standardized factor loadings (<0.50); four items (‘I_3–I_4’, ‘SI_1–SI_2’) were removed due to high residual covariances. The assessment results of the measurement model reached a marginally adequate fit (χ^2^/*df* = 1.676, RMSEA = 0.077, CFI = 0.871, TLI = 0.858, SRMR = 0.078). Although the CFI and TLI remained slightly below the traditional 0.90 threshold, the acceptable RMSEA and SRMR values (<0.08), combined with the robust CR and AVE, suggest that the measurement model was sufficiently reliable for the subsequent exploratory structural analyses. Importantly, following the removal of negatively worded items, the revised “Emotions” measure represented predominantly positive emotional experience (e.g., happy). Therefore, the structural effects of this construct in our analyses should be interpreted strictly as the facilitative role of positive emotions. Further details can be found in Table A2 of Appendix A.

### 3.2. Structural Equation Modeling

SEM was used to examine the hypothesized relations among variables while employing maximum likelihood estimation.

**Figure A1:**
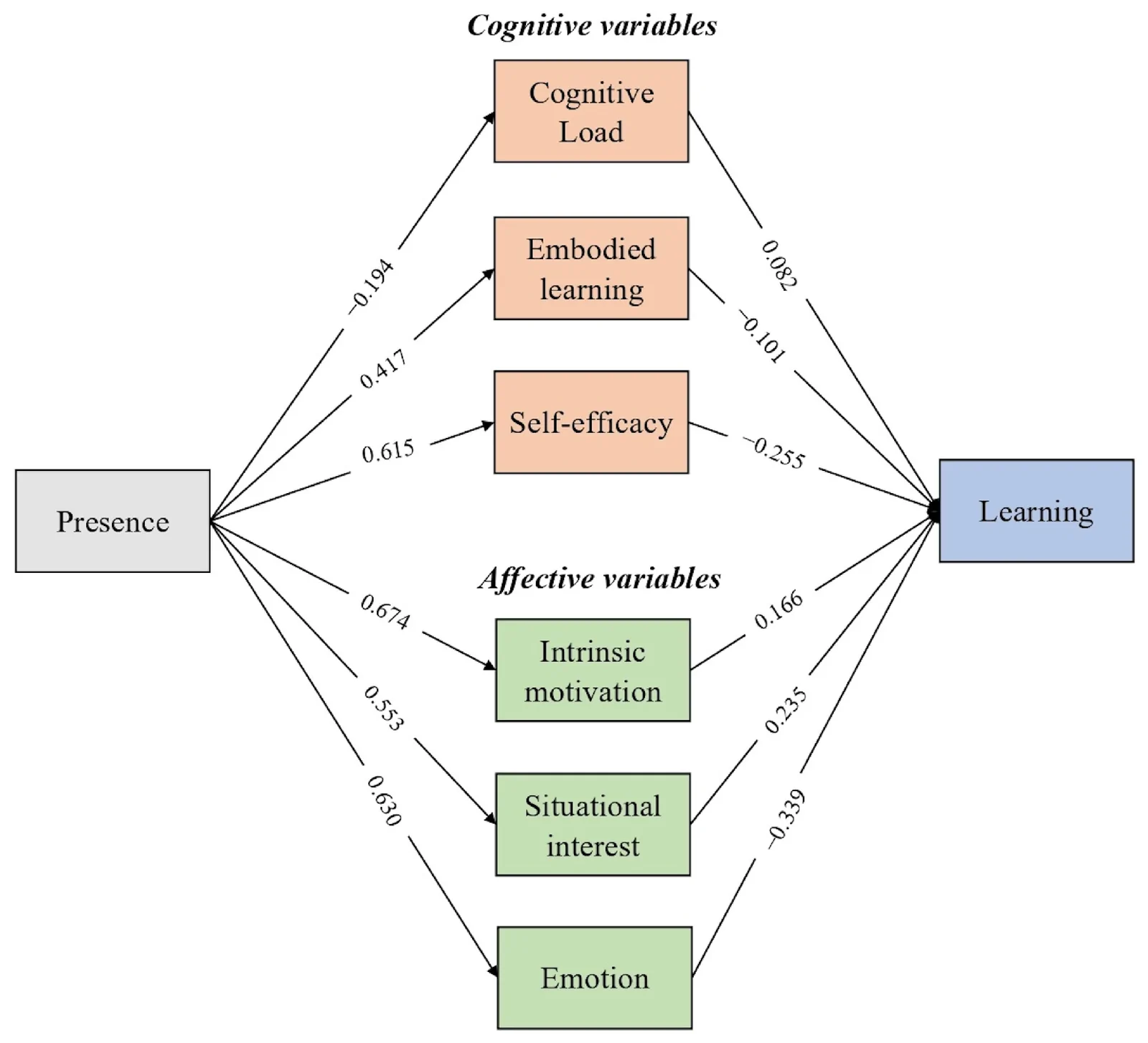
The initial model on reaction times. *Note*: Arrows indicate the direction of predictive relationships between variables.

Confirmatory analyses: The initial SEM aimed to test the a priori CAMIL model. This model’s fit was not acceptable at first (RMSEA = 0.328, CFI = 0.561, TLI = 0.232, SRMR = 0.151) (see details in Figure A1 of Appendix A).

**Figure 3:**
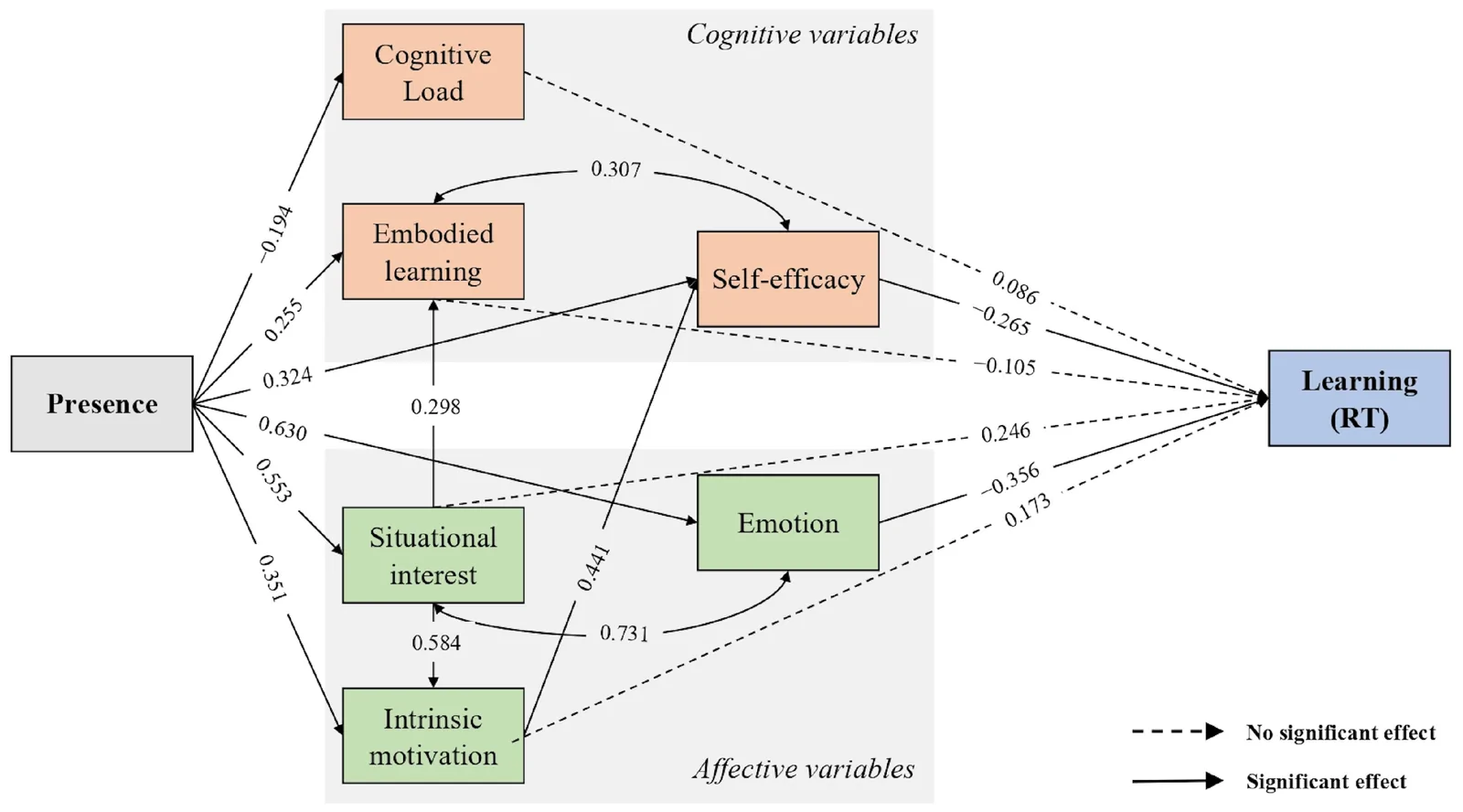
The final model on reaction times. *Notes*: Arrows indicate the direction of predictive relationships between variables: Solid arrows represent statistically significant effects, and dashed arrows represent non-significant effects. Numbers displayed within the arrows are standardized path coefficients.

Exploratory analyses: Given the model’s poor initial fit, we proceeded with an exploratory, data-driven approach to modify the model. Modification indices were used to adjust the model based on the data. This resulted in the addition of covariance between the following items from the same scales (not in the graphical illustration of the final model): situational interest and emotions, embodied learning, and self-efficacy. Additionally, paths were added between the following observed variables (shown in the final model): self-efficacy and intrinsic motivation, intrinsic motivation and situational interest, embodied learning, and situational interest. All adjustments based on modification indices were psychometrically and theoretically sound (for more details, see Section 4). These adjustments resulted in the simplified model presented in Figure 3, which obtained an acceptable fit (RMSEA = 0.045, CFI = 0.994, TLI = 0.985, SRMR = 0.045).

#### 3.2.1. Hypothesis 1: Presence Will Predict Cognitive Variables (Cognitive Load, Embodied Learning, and Self-Efficacy)

Hypothesis 1 was supported: Presence predicted all three cognitive factors: Cognitive load (*β* = −0.194, *p* = 0.044), embodied learning (*β* = 0.255, *p* = 0.012), and self-efficacy (*β* = 0.324, *p* < 0.001).

#### 3.2.2. Hypothesis 2: Presence Will Predict Affective Variables (Situational Interest, Intrinsic Motivation, and Emotions)

Hypothesis 2 was supported: Presence predicted all three affective factors: Intrinsic motivation (*β* = 0.351, *p* < 0.001), situational interest (*β* = 0.553, *p* < 0.001), and emotions (*β* = 0.630, *p* < 0.001).

#### 3.2.3. Hypothesis 3: Cognitive Variables Will Predict Learning

Hypothesis 3 was partially supported: Learning was significantly predicted by self-efficacy (*β* = −0.265, *p* = 0.043) but was neither related to cognitive load (*β* = 0.086, *p* = 0.372) nor embodied learning (*β* = −0.105, *p* = 0.347).

#### 3.2.4. Hypothesis 4: Affective Variables Will Predict Learning

Hypothesis 4 was partially supported: Learning was significantly predicted by emotions (*β* = −0.356, *p* = 0.031) but not by intrinsic motivation (*β* = 0.173, *p* = 0.307) or situational interest (*β* = 0.246, *p* = 0.182).

#### 3.2.5. Exploratory Findings

Situational interest predicted embodied learning (*β* = 0.298, *p* = 0.003) and intrinsic motivation (*β* = 0.584, *p* < 0.001). Intrinsic motivation predicted self-efficacy (*β* = 0.441, *p* < 0.001). Furthermore, there was covariance between emotions and situational interest (*β* = 0.731, *p* < 0.001), as well as between embodied learning and self-efficacy (*β* = 0.307, *p* = 0.001).

## 4. Discussion

In the context of the CAMIL, this study investigated the process of receptive L2 vocabulary learning through IVR. Using SEM, we examined the influence of IVR presence on affective and cognitive factors, as well as their subsequent effects on outcomes of L2 vocabulary recognition. Below, we discuss the empirical, theoretical, and practical implications and contributions of our findings.

### 4.1. Cognitive and Affective Paths from IVR Presence to L2 Vocabulary Learning

While presence predicted the cognitive variables of cognitive load, self-efficacy, and embodied learning, as well as the affective variables of situational interest, intrinsic motivation, and emotions, as expected, only self-efficacy and emotions predicted L2 recognition performance. As shown in Figure 3, both patterns of association from presence to L2 recognition performance are labeled as cognitive and affective paths, which form the major empirical contributions of the present study. It is worth noting that the negative coefficients observed from self-efficacy and emotions paths to learning suggest a facilitative effect such that higher efficacy and positive emotions yielded more efficient lexical processing, as shown by faster RTs.

The first significant path was the cognitive path in which L2 vocabulary recognition performance was positively predicted by level of self-efficacy. Self-efficacy, in turn, was predicted by participants’ level of presence. These results partly align with findings reported by Petersen et al. (2022), who investigated the process of learning a science lesson on viral diseases using IVR. The authors found that self-efficacy, but not presence, predicted learning. We argue that the different findings between Petersen et al. (2022) and our study might arise from the difference in learning materials (i.e., science vs. language learning). Learning different types of knowledge through IVR may lead to distinct interactions across factors (e.g., self-efficacy) that are related to learning. Moreover, the findings in our study are in line with previous work on classroom-based learning (e.g., Khodadad & Kaur, 2016) and remote learning (e.g., Teng et al., 2023), contexts in which self-efficacy predicted L2 learning outcomes.

The second significant path was the affective path in which L2 vocabulary recognition performance was positively predicted by participants’ degree of emotions. Emotions, again, were predicted by participants’ level of presence. These results echo findings reported by Parong and Mayer (2018, 2021), who found that biology lessons learned through IVR, compared to a slideshow format, led to increased positive emotions, and that these increases predicted learning outcomes. Our findings are also consistent with previous research on multimedia learning, suggesting that the emotional design of a multimedia lesson can induce positive emotions in learners, which, in turn, facilitate comprehension and learning (Mayer & Estrella, 2014; Plass et al., 2014). Moreover, the affective path in the present study corroborates previous work on emotions and memory, suggesting that positive emotions facilitate encoding during learning and help retrieval of information (see Tyng et al., 2017 for a review).

### 4.2. Sequences of Associations from IVR Presence to L2 Vocabulary Learning

Beyond the significant structural associations of self-efficacy and emotions, additional patterns of associations linking presence to receptive L2 vocabulary learning were identified. First, although situational interest did not directly predict L2 recognition performance, we found a sequential pattern between the two which intersected with intrinsic motivation and self-efficacy. We also found a path between intrinsic motivation and receptive L2 vocabulary processing which intersected with self-efficacy. This is consistent with findings from a study by Makransky and Petersen (2019) in which the authors investigated the process of learning about medical genetics using desktop virtual reality. The authors found that presence predicted intrinsic motivation, and that intrinsic motivation predicted self-efficacy, leading to increased learning outcomes. Moreover, our results are similar to Makransky et al.’s (2020a) findings showing that immersive simulations with integrated assessment and feedback increased students’ intrinsic motivation, self-efficacy, and learning. Analogous patterns have been observed in L2 vocabulary learning, in which virtual reality facilitated learners’ self-efficacy and motivation during the learning process (Y. J. Lin & Wang, 2021). However, these studies have only focused on how immersive simulations affect intrinsic motivation, self-efficacy, and learning outcomes. By examining the relationships across these variables during the learning process, our findings add to this growing body of knowledge by uncovering the pathways through which the variables differentially contribute to receptive L2 vocabulary processing.

While some cognitive and affective pathways were identified through which IVR presence influenced receptive L2 vocabulary processing, certain variables—such as cognitive load and embodied learning—neither directly nor indirectly predicted learning outcomes. These null results may be attributed to limitations in our IVR design, which might not have elicited a sufficient level of cognitive load or a strong enough embodied learning experience. Another possible explanation is the limited sensitivity of the self-report measures used to assess these constructs, which may have constrained our ability to detect their nuanced effects.

Overall, our findings show how IVR contributes to receptive L2 vocabulary recognition and extend the existing literature on learning through IVR environments. While previous IVR studies have reported on the process of science learning (e.g., learning about viral diseases or genetics), the present study investigated how IVR contributes to the process of L2 vocabulary recognition, adding to the scant body of work examining this topic.

### 4.3. Theoretical Contributions

From a language learning perspective, the present work has provided an initial empirical exploration of how IVR contributes to receptive L2 vocabulary learning as contextualized through the CAMIL (Makransky & Petersen, 2021). By using SEM, our analyses and findings have elucidated the process of receptive vocabulary learning in IVR by identifying relationships between affective and cognitive factors. While our findings offer support for the CAMIL’s hypothesis that presence is a predictor of cognitive and affective factors, we have also found that it was only related to learning via a sequence of associations involving self-efficacy and emotions. Additionally, the recognition performance of L2 vocabulary exhibited a sequential pattern of associations with situational interest and intrinsic motivation, providing further, albeit structural, support for the CAMIL.

These findings generate theoretically meaningful patterns that could inform future research. For instance, our results showed that situational interest predicted embodied learning, not the other way around as suggested by the CAMIL. A possible explanation for this observation might be that when learners are interested in a given situation, they are more likely to actively participate and fully engage themselves (Hidi & Renninger, 2006). Moreover, the results supported an association between embodied learning and self-efficacy during the cognitive process, as well as a link between situational interest and emotions during the affective process. These connections have previously been discussed in research on learning (e.g., Petersen et al., 2022). Overall, our findings demonstrate that there are complex cognitive-affective interactions in the process of L2 vocabulary learning that require additional, independent confirmation in future work.

### 4.4. Limitations and Future Directions

This study tested a framework for receptive L2 vocabulary learning through IVR. Given that there are several variables that may play a role in this learning process, future research should explore other factors that were not included in the a priori model that we tested (e.g., executive function; Lawson & Mayer, 2024). Furthermore, we must acknowledge that the final model (refer back to Figure 3) included data-driven modifications to achieve an acceptable fit. Although we strictly separated our confirmatory and exploratory analyses and provided strong theoretical justifications for these modifications, the risk of statistical overfitting cannot be entirely ruled out. Future studies should aim to cross-validate this modified model using independent and larger samples.

Another limitation of the present study pertains to the target language and the amount of vocabulary learned by the participants. For instance, despite our efforts to ensure that participants had no prior knowledge of Korean, the peculiarities of Korean as an L2 for beginners may have uniquely influenced the participants’ cognitive load and learning efficiency during learning. Moreover, compared to real-world L2 learning, a small set of 20 words may limit the ecological validity of the findings. Therefore, future studies should aim to have larger, more ecological vocabulary sets and various L2s to determine the generalizability of our findings.

Moreover, it is important to acknowledge the limitations of the lexical recognition task that we used to measure learning outcomes. This particular task primarily assesses receptive knowledge and initial L2 form-meaning mapping. In order to build a more holistic understanding of IVR-based language learning, future research should incorporate productive measures, such as picture naming, to evaluate learners’ ability to actively produce L2 words. Finally, potential confounding factors such as participants’ prior experience with IVR, general familiarity with digital tools, and possible cultural biases were not systematically controlled or assessed. Subsequent work should consider incorporating a control group and using traditional learning methods to help control for potential effects of these confounding factors and to validate the unique effects of IVR on L2 learning.

## 5. Conclusions

This study sheds light on the complex interplay between IVR presence, affective and cognitive variables, and L2 vocabulary recognition performance. Presence was found to contribute significantly to the affective and cognitive paths by increasing learners’ sense of emotions and self-efficacy, which led to increased efficiency in L2 recognition. While these exploratory structural patterns require further confirmation, they add to our understanding of the underlying processes by which IVR influences L2 vocabulary recognition. By elucidating these patterns underlying receptive language learning in immersive environments, our findings provide valuable insight for researchers and practitioners in the field of language teaching and learning.

## Figures and Tables

**Table 1:** Scale quality.

Construct	Cronbach’s α
Presence	0.82
Cognitive load	0.91
Embodied learning	0.81
Situational interest	0.91
Intrinsic motivation	0.90
Self-efficacy	0.84
Emotions	0.82

## Data Availability

The data in this study are available from the corresponding authors upon reasonable request.

## Appendix A

**Table A1:** Survey items and sources.

Construct	Item Code	Items	Chinese Version	Source
Presence	P_1	The virtual environment seemed real to me.	我觉得虚拟现实场景很真实。	Makransky et al. (2017)
	P_2	I had a sense of acting in the virtual environment, rather than operating something from outside.	我有一种在虚拟环境中行动的感觉，而不是觉得自己是在外部进行操作。	
	P_3	My experience in the virtual environment seemed consistent with my experiences in the real world.	我在虚拟环境中的体验和在现实世界中的体验是一致的。	
	P_4	I had a sense of “being there” in the virtual environment.	我在虚拟环境中有一种“置身其中”的感觉。	
	P_5	I was completely captivated by the virtual world.	虚拟世界让我深深着迷。	
	P_6	I felt like I was immersed (or included in) and interacting with the computer environment,	您能够在多大程度上感觉自己沉浸在（或包含在）计算机环境中并与之互动	Witmer and Singer (1998)
	P_7	How completely were you able to actively survey or search the environment using vision?	您能够在多大程度上充分利用视觉来积极地调查或搜索环境?	
Cognitive load	C_1	The learning content of this activity was very complex.	这项活动的学习内容非常复杂。	Leppink et al. (2014)
	C_2	In this activity, very complex terms were mentioned.	这项活动里提到了非常复杂的术语。	
	C_3	I invested a very high mental effort in the complexity of this activity.	这项活动太复杂，让我投入了很多的脑力。	
	C_4	The explanations and instructions in this activity were very unclear.	这项活动中的说明和指示非常含混难懂。	
	C_5	The explanations and instructions in this activity were full of unclear language.	这项活动中的说明和指示充满了含混难懂的语言。	
	C_6	The explanations and instructions in this activity were, in terms of learning, very ineffective.	这项活动中的说明和指示对学习非常无效。	
	C_7	I invested a very high mental effort in unclear and ineffective explanations and instructions in this activity.	说明和指示的含混难懂让我在这项活动中投入了很多的脑力。	
Embodied learning	EL_1	During the lesson, my movements and bodily activities were an integrated part of learning.	在课程中，我的动作和身体活动是学习的一个组成部分。	Skulmowski and Rey (2018)
	EL_2	During the lesson, I performed bodily movements and interactions that were relevant for learning.	在课程中，我进行了与学习相关的身体运动和互动。	
	EL_3	Performing gestures/movements during the lesson helped me learn.	在课程中利用手势或者其他动作帮助我学习。	
Situational interest	SI_1	Did the lesson spark your curiosity?	该学习过程激发了你的好奇心吗？	Knogler et al. (2015)
	SI_2	Did the lesson capture your attention?	该学习过程引起你的注意了吗？	
	SI_3	Were you concentrated on the lesson?	该学习过程中你专心学习了吗？	
	SI_4	Was the lesson entertaining for you?	该学习过程对你来说有趣吗？	
	SI_5	Did you have fun during the lesson?	该学习过程中你玩得开心吗？	
	SI_6	Was the lesson exciting for you?	该学习过程让你兴奋吗？	
Intrinsic motivation	I_1	I enjoy working on experimental projects on virtual reality scenarios.	我非常喜欢参加虚拟现实场景下的实验项目。	Makransky and Petersen (2019)
	I_2	It’s fun to learn vocabulary in a virtual reality scenario.	在虚拟现实场景下学习词汇很有趣。	
	I_3	Vocabulary learning is boring in a virtual reality scenario. (R)	在虚拟现实场景下学习词汇很无聊。	
	I_4	Virtual reality lab doesn’t appeal to me at all. (R)	虚拟现实实验室对我一点吸引力没有。	
	I_5	I would describe the virtual experiment as very interesting.	我认为虚拟现实场景的实验项目非常有趣。	
	I_6	I thought that the virtual experiment was quite enjoyable	我认为虚拟现实场景的实验项目令人非常愉悦。	
	I_7	While I was doing the virtual experiment, I was thinking about how much I enjoyed it.	当我参加虚拟现实场景下的实验项目时，我会思考自己是否很享受其中。	
	I_8	I felt motivated to learn the vocabulary	我感到有动力去学习词汇。	Parong and Mayer (2021)
	I_9	I found vocabulary learning to be useful to me	我认为学习词汇对我很有用。	
Self-efficacy	S_1	I’m confident I can remember the vocabulary.	我相信我能学会这些词汇。	Meyer et al. (2019)
	S_2	I’m confident I can remember the most complex vocabulary.	我相信我能学会最复杂的词汇。	Pintrich et al. (1991)
	S_3	I’m confident I could do an excellent job on vocabulary learning.	我相信我可以出色地完成词汇学习。	
	S_4	I’m confident I could recall the learned vocabulary correctly.	我相信我可以正确回忆学过的词汇。	
Emotions	E_1	I felt happy during the lesson.	我在学习过程中感到快乐。	Parong and Mayer (2018)
	E_2	I felt excited during the lesson.	我在学习过程中感到兴奋。	
	E_3	I felt bored during the lesson. (R)	我在学习过程中感到无聊。	
	E_4	I felt confused during the lesson. (R)	我在学习过程中感到困惑。	
	E_5	I felt sad during the lesson. (R)	我在学习过程中感到悲伤。	

**Table A2:** Original and revised measurement properties.

Construct	Original			Revised		
	Items	AVE	CR	Items	AVE	CR
Presence	7	0.44	0.85	7	0.44	0.84
Cognitive load	7	0.61	0.91	7	0.61	0.91
Embodied learning	3	0.64	0.84	3	0.64	0.84
Situational interest	6	0.65	0.92	4	0.66	0.89
Intrinsic motivation	9	0.53	0.91	7	0.56	0.90
Self-efficacy	4	0.57	0.84	4	0.57	0.84
Emotions	5	0.48	0.81	3	0.66	0.85

Note: AVE = Average Variance Extracted; CR = Composite Reliability.
